# *Lycium barbarum* Polysaccharide Suppresses Expression of Fibrotic Proteins in Primary Human Corneal Fibroblasts

**DOI:** 10.3390/jcm9113572

**Published:** 2020-11-06

**Authors:** Sum Sum Kwok, Francisca Siu-Yin Wong, Kendrick Co Shih, Yau-Kei Chan, Yashan Bu, Tommy Chung-Yan Chan, Alex Lap-Ki Ng, Amy Cheuk-Yin Lo, Louis Tong, Gary Hin-Fai Yam, Vishal Jhanji

**Affiliations:** 1 Department of Ophthalmology, Li Ka Shing Faculty of Medicine, University of Hong Kong and Hong Kong SAR, Hong Kong, China; suzie227@hku.hk (S.S.K.); frannwong@gmail.com (F.S.-Y.W.); josephyk@connect.hku.hk (Y.-K.C.); u3005204@connect.hku.hk (Y.B.); tommychan.me@gmail.com (T.C.-Y.C.); lkalex@gmail.com (A.L.-K.N.); amylo@hku.hk (A.C.-Y.L.); 2 Cornea and External Eye Disease Service, Singapore National Eye Centre, Singapore 168751, Singapore; louis.tong.h.t@singhealth.com.sg; 3 Ocular Surface Research Group, Singapore Eye Research Institute, Singapore 169856, Singapore; 4 Department of Ophthalmology, University of Pittsburgh Medical Centre, Pittsburgh, PA 15213, USA; gary.yam@pitt.edu (G.H.-F.Y.); vishaljhanji@cuhk.edu.hk (V.J.)

**Keywords:** *Lycium barbarum* polysaccharides, corneal scarring, human keratocytes, fibroblast, TGFβ1

## Abstract

(1) Objective: To study the anti-fibrotic effects of *Lycium barbarum* polysaccharides (LBP) on corneal stromal fibroblasts and assess LBP’s effect on cell viability. (2) Methods: Primary human corneal keratocytes of passage 3 to 6 were used for all experiments. Cells are pretreated with LBP solution for 24 h and then transforming growth factor beta 1 (TGFβ1) for 48 h and collected for experiments. Fibrotic protein analysis was performed using immunofluorescence and Western blot. The effect of LBP on cell viability was assessed using the MTS assay. (3) Results: LBP significantly reduced the expression of fibrotic proteins, including α-SMA and extracellular matrix proteins (collagen type I and III). LBP significantly decreased the viability of myofibroblasts but not the fibroblasts. Conclusions: In this study, LBP was effective in the prevention of fibrosis gene expression. Further studies to assess the underlying mechanism and pharmacological properties will facilitate the formation of a topical LBP solution for in vivo studies.

## 1. Introduction

Corneal scarring is the second most common cause of blindness worldwide [1]. Definitive treatment of corneal scarring requires corneal transplantation which is limited by low organ donation rates, especially in the developing world. Currently, there are no treatments available to prevent corneal scarring [2]. Corneal scarring is often due to infection or injury [3]. Other causes include corneal dystrophies, corneal ectasia, or inflammatory diseases, such as allergies or ocular rosacea [4,5].

Upon disruption of the basal epithelium and stromal layers, inflammatory cytokines and fibrogenic growth factors, such as transforming growth factor-β1 (TGFβ1), transforming growth factor-β2 (TGFβ2), and connective tissue growth factor (CTGF) are released from the epithelium [6]. TGFβ1 is a key cytokine in promoting myofibroblast differentiation with increased production of disorganized stromal matrix proteins, including collagen, fibronectin, tenascin, proteoglycans, thrombospondin and tissue inhibitor of metalloprotease-1 (TIMP-1). This can lead to increased light scattering due to development of corneal haze and scarring [7]. TGFβ1 signaling is mediated via Smad-dependent and non-dependent pathways which regulate various fibrosis gene expressions. Smad2, Smad3 and Smad4 are pro-fibrotic while Smad6 and Smad7 are anti-fibrotic [7]. TGFβ2 plays a major role in corneal development specifically the epithelial-mesenchymal interactions, corneal homeostasis and repair [8]. Current treatments such as topical corticosteroids are associated with potentially blinding complications, such as cataract, glaucoma and cornea-scleral melting [2]. Topical mitomycin C has shown to commonly induce allergic reactions and punctal stenosis [9]. There are reports on sight-threatening complications of topical mitomycin C, including iritis, corneal perforation, scleral calcification, incapacitating photophobia and pain and corneal edema [10].

Various topical and systemic treatments have been studied in the prevention and minimization of cornea scarring after injury. Topical steroids and mitomycin C are currently used in clinical practice to reduce corneal haze after refractive surgery and minimize cornea scarring in keratitis, respectively. However, in the case of topical steroid use, there is a significant risk of exacerbating infectious keratitis as a result of certain microbes, including Nocardia species, atypical mycobacteria and fungi. In the case of mitomycin C, its use in cornea disease beyond preventing haze after laser refractive surgery remains unproven [2]. Furthermore, there are a number of emerging therapies with promising results in laboratory-based studies and small-scale clinical trials. For example, topical rosiglitazone which has been shown in vivo to be able to reduce α-SMA immunostaining after photorefractive keratectomy (PRK), thus allowing the cornea to return to its original state of clarity and refractive properties after 3 months of treatment without any major side effects [11]. In addition, intake of systemic vitamin C has been shown to result in smaller epithelial defect size and decreased corneal haze density in patients with infectious keratitis [12]. A number of studies have investigated the use of gene therapy in reducing corneal haze after injury. Triple small interfering RNA (siRNA) therapy, targeting pro-fibrosis genes including TGFβ, TGFb receptor (TGFBR2), and CTGF, was compared to prednisolone using an ex vivo model in one experiment. The study found that there was a 77% and 85% reduction in SMA expression in siRNA treated groups and prednisolone treated groups respectively, thus demonstrating that siRNA therapy may have similar efficacy to prednisolone [6]. Furthermore, targeted delivery of Smad7 using recombinant adeno-associated virus serotype 5 (AAV5-Smad7) to the cornea immediately after PRK significantly reduced the presence of α-SMA. This corresponded with a reduction in cornea cloudiness and haze in AAV5-Smad7-treated eyes. There were no significant adverse effects detected, including raised intraocular pressure and cornea epithelial toxicity [13]. Notably, due to their current availability, good safety profiles and relatively low costs, systemic vitamin C and topical rosiglitazone may serve as more viable forms of therapy, compared to gene therapy, in the near future.

*Lycium barbarum* polysaccharides (LBP) are extracted from goji berries or wolfberries. They are a group of sugars including glucose, arabinose, galactose, mannose, rhamnose and xylose [14]. Studies have shown that dietary administration of LBP to rats with non-alcoholic steatohepatitis (NASH) showed significantly less hepatic fibrosis than the control group [15]. LBP has also been shown to prevent hepatic fibrosis from direct toxicity by carbon tetrachloride (CCl4) in rats, with a clear negative dose-response relationship between LBP concentration and hepatic enzymes alanine transferase (ALT) and aspartate transferase (AST) [16]. In an in vitro study on cornea epithelial cells, LBP significantly reduced UV-B light related damage to corneal epithelial cells through inhibition of the JNK-Bax-caspase 3 pathway. This pathway has been implicated in the pathogenesis of cornea stromal fibrosis [17]. Given the strong evidence showing LBP’s efficacy in reducing liver fibrosis after injury, the close similarities in cell biology between hepatic fibroblasts and cornea fibroblasts, and LBP’s good safety profile and low cost, LBP may prove to be a viable topical anti-fibrotic agent in cornea epithelial-stromal injury. We conducted a proof-of-concept experiment using primary corneal fibroblasts.

## 2. Materials and Methods

### 2.1. Cell Culture

Primary human corneal fibroblasts were obtained from ScienCell Research Laboratories (San Diego, CA, USA). The cells were cultured using fibroblast medium, supplemented with 2% fetal bovine serum (FBS) and fibroblast growth supplement (all reagents were supplied by ScienCell Res. Lab.). They were cultured at 37 °C and 5% CO_2_ balanced with air. Cells at passage 3 to 6 were used in all experiments.

### 2.2. Cell Characterization

The fibroblasts were characterized based on vimentin and fibronectin expression using immunocytochemistry as well as fibroblast-like morphology under phase contrast microscopy.

### 2.3. LBP Preparation and Treatment

LBP powder with 82% polysaccharide content (XABC Biotech, Xian, China) was dissolved in Dulbecco’s phosphate buffered saline (DPBS) at a concentration of 10 mg/mL. After filter-sterilization (0.22 µm minipore filter; Merck KGaA, Darmstadt, Germany), it was diluted in fibroblast culture medium for cell treatment.

### 2.4. Treatment of Cells

Once cells reached 30% confluence, cells were pre-treated with LBP at 0.1 mg/mL, 0.5 mg/mL, 1 mg/mL, 1.5 mg/mL, 2.0 mg/mL, 2.5 mg/mL or 3.0 mg/mL treated for 24 h and then with TGFβ1 (Millipore, GF439, Burlington, MA, USA) at 10 ng/mL for 48 h to differentiate the cells into myofibroblasts.

### 2.5. Immunofluorescence

Cells were cultured on poly-L-lysine coated glass coverslips until around 80% confluence. They were washed with DPBS and then fixed with 4% paraformaldehyde for 10 min. After washing with ice-cold phosphate buffered saline (PBS) for 3 times, they were permeabilized with 0.1% Triton X-100 in PBS for 10 min and washed with PBS 3 times for 5 min each. The cells were blocked with 1% bovine serum albumin (BSA) in PBST for 30 min. Incubation of primary antibodies (Table 1) was done in 1% BSA at 4 °C overnight, followed by washes with PBS, 3 times for 5 min each. Secondary antibody incubation was done in 1% BSA for 1 h at room temperature and washed with PBS 3 times for 5 min each. Nuclear staining was done with Hoechst 33,342 (ThermoFisher, H3570, Waltham, MA, USA) at 1:1500 dilution in PBS for 5 min. The cells were washed with PBS and mounted in aqueous mounting medium (Abcam, ab128982, Cambridge, UK) for imaging.

### 2.6. Western Blot

Cells after treatment were trypsinized and cell pellets were lysed with mammalian protein extraction reagent (MPER) (ThermoFisher, # 78501) supplemented with protease inhibitors EDTA (ThermoFisher, #78438 for both) and phosphatase inhibitor (ThermoFisher, #78427). Protein quantification was done using the BCA assay (ThermoFisher, #23225) according to the manufacturer’s instructions. For this, 12.5% polyacrylamide gels were used. An equal amount of protein was loaded into each well (15 µg) and transferred onto polyvinylidene difluoride (PVDF) membranes. Blots were blocked with 5% non-fat milk in Tris-buffered saline with 0.1% Tween 20 (TBST) for 1 h at room temperature. Incubation of primary antibodies (Table 2) was done at 4 °C overnight. Blots were washed 3 times with TBST and incubated with species-specific horseradish peroxidase (HRP) conjugated secondary antibodies for 1 h at room temperature. Blots were washed with TBST 3 times and visualized using the chemiluminescence (Amersham ECL Western Blotting Detection Reagent; GE Healthcare, Life Sciences, #RPN2106). ImageJ software (Version 1.80_72, National Institutes of Health, Bethesda, MD, USA) was used to determine the band intensity by plotting histograms of individual bands and calculating the area under curve to calculate protein concentration. These protein concentrations were then normalized with GAPDH. All experiments were repeated 5 times.

### 2.7. Cell Viability Assay

A total of 1500 cells were seeded into each well of a 48-well plate for the MTS assay (Promega, G5421, Promega, Madison, WI, USA). The MTS test was done according to the manufacturer’s instructions. The cells were incubated in the MTS reagents for 75 min in an incubator at 37 °C and 5% CO2. The MTS assays were done separately on fibroblasts with LBP treatment alone and fibroblasts pre-treated to LBP solution and then to TGF-β1 to compare the effect LBP on the viability of fibroblasts and myofibroblasts separately. All experiments were repeated 5 times.

### 2.8. Statistical Analysis

Statistical analysis was done using GraphPad Prism 7 (Version 7, GraphPhad Software Inc, San Diego, CA, USA) using One-Way Analysis of Variance (ANOVA). All data are presented as mean ± standard deviation.

## 3. Results

### 3.1. Corneal Fibroblasts Express Vimentin and Fibronectin

**Figure 1:**
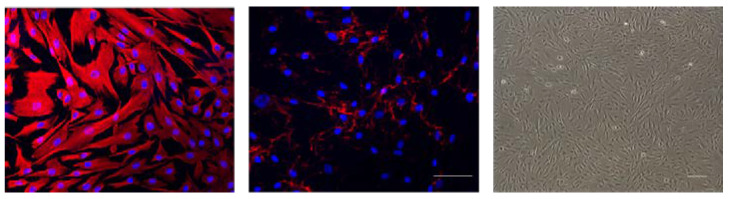
Immunofluorescence images showing vimentin expression (red, left) and fibronectin expression (red, middle) and nuclear staining with Hoechst 33,342 (blue) along with phase-contrast image showing fibroblast morphology (right). Scale bar = 100 µm.

Immunofluorescence images showed strong vimentin expression of cells and moderate levels of fibronectin expression (Figure 1). Phase-contrast microscopy demonstrated bipolar-elongated fibroblast-like morphology of the cells (Figure 1).

### 3.2. LBP Selectively Affects Myofibroblast Cell Viability

**Figure 2:**
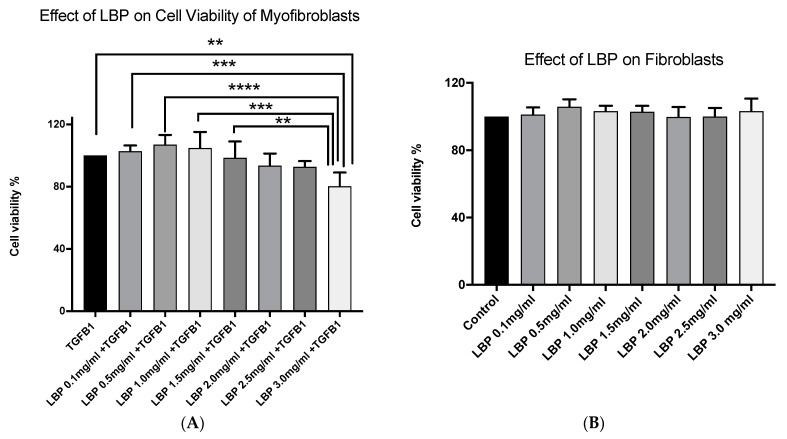
Graphs showing the effect of *Lycium barbarum* polysaccharides (LBP) on myofibroblasts (**A**) (*n* = 5 for each group) vs. fibroblasts (**B**) (*n* = 5 for each group) using one-way ANOVA, ** *p* < 0.01, *** *p* < 0.001, **** *p* < 0.0001.

We compared the effect of LBP in both myofibroblasts and fibroblasts. The trend of effect of LBP on myofibroblasts was statistically significant (*p* < 0.0001) (Figure 2A). There was a also a dose-dependent reduction in cell viability of LBP 1.5 mg/mL, LBP 2.0 mg/mL, LBP 2.5 mg/mL and LBP 3.0 mg/mL treatment group by 1.4%, 6.4%, 7.1% and 19.7% respectively, with the LBP 3.0 mg/mL treatment group being the only one with statistically significant reduction of cell viability compared to TGFβ1 treatment alone (*p* = 0.0034) (Figure 2A). For myofibroblasts treated with LBP 0.1 mg/mL, LBP 0.5 mg/mL and LBP 1.0 mg/mL there was an increase in cell viability from 2.9%, 7.1% and 5.0%, respectively. There was no statistically significant effect on fibroblasts (*p* = 0.4341) (Figure 2B). For LBP0.1 mg/mL, LBP 0.5 mg/mL, LBP 1.0 mg/mL, LBP 1.5 mg/mL, LBP 2.5 mg/mL and LBP 3.0 mg/mL there was an increase of cell viability by 1.2%, 5.8%, 3.1%, 2.8%, 0.084% and 3.2%, respectively. Only LBP 2.0 mg/mL showed a reduction in cell viability of 0.2% but this was not statistically significant.

### 3.3. LBP Attenuates TGFβ1-Induced Fibrosis

**Figure 3:**
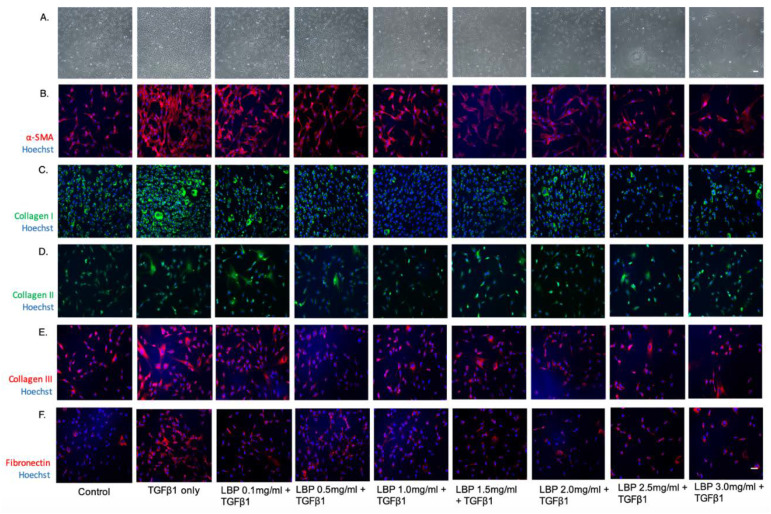
Immunofluorescence images showing the effect of LBP on α-SMA (**B**), collagen type I (**C**), collagen type II (**D**) and collagen type III (**E**) and fibronectin (**F**) expression along with phase contrast microscopy images (**A**). Nuclear staining was done with Hoechst 33,342 (blue). Scale bar = 100 µm.

Cells treated with TGFβ1 showed increased α-SMA expression, a marker for myofibroblast differentiation, compared to control (Figure 3). LBP treatment resulted in dose-dependent reduction of α-SMA expression (Figure 3). TGFB1 treatment resulted in a trend of increased Collagen type 1 expression and a notable trend of reduced Collagen type 1 expression with LBP treatment (Figure 3). Collagen type II expression showed no significant trend with LBP treatment (Figure 3). TGFβ1-treated cells also showed a trend of increased Collagen type III expression and reduction with LBP treatment (Figure 3). It is also possible that LBP pre-treatment reduces cell proliferation based on the trend of dose-dependent reduction in cell density.

**Figure 4:**
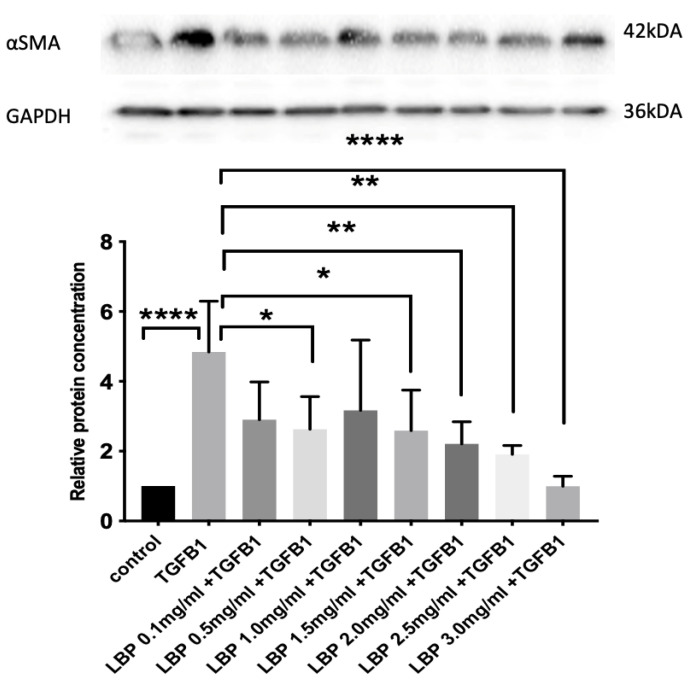
Graph showing α-SMA protein quantification (bottom) with Western blot (top) showing the effect of LBP on α-SMA concentration using one-way ANOVA (*n* = 5 for each group), **** *p* < 0.0001, ** *p* < 0.01, * *p* < 0.05.

Western blot showed that treatment of fibroblasts with TGFβ1 alone resulted in nearly 4 times increase in α-SMA expression compared to the control (Figure 4). Treatment with LBP at 0.5 mg/mL, 1.5 mg/mL, 2.0 mg/mL, 2.5 mg/mL and 3.0 mg/mL resulted in statistically significant reduction in α-SMA expression (*p* = 0.0489, *p* = 0.0424, *p* = 0.0097, *p* = 0.0028 and *p* < 0.0001, respectively) compared to the TGFβ1 alone group. The concentration of α-SMA were reduced by 34.5% to 79.4%, after TGFβ1 treatment (Figure 4).

### 3.4. LBP Has No Significant Effect on Fibroblasts

**Figure 5:**
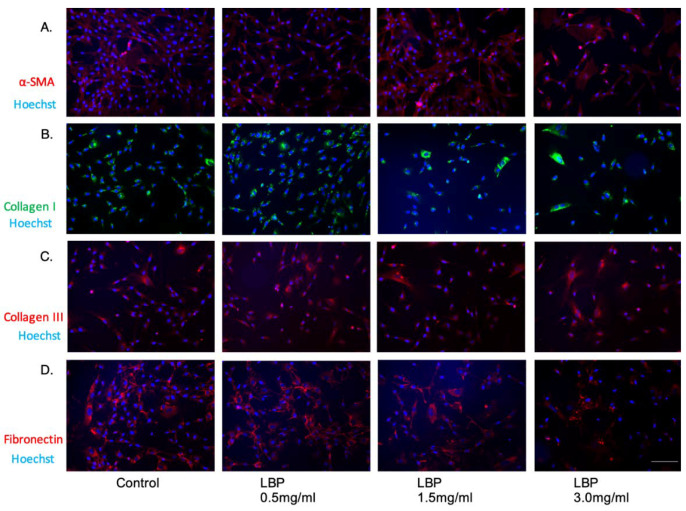
Immunofluorescence images of the negative controls showing the effect of LBP on α-SMA (**A**), collagen type I (**B**), and collagen type III (**C**) and fibronectin (**D**) expression. Nuclear staining was done with Hoechst 33,342 (blue). Scale bar = 100 µm.

We also assessed the effect of LBP on fibroblasts at LBP 0.5 mg/mL, LBP 1.5 mg/mL and LBP 3.0 mg/mL (Figure 5). There was no noticeable effect of LBP on α-SMA, collagen type I and collagen type III expression (Figure 5). However, there was an observable reduction in fibronectin expression with LBP 3.0 mg/mL treatment compared to the control (Figure 5).

**Figure 6:**
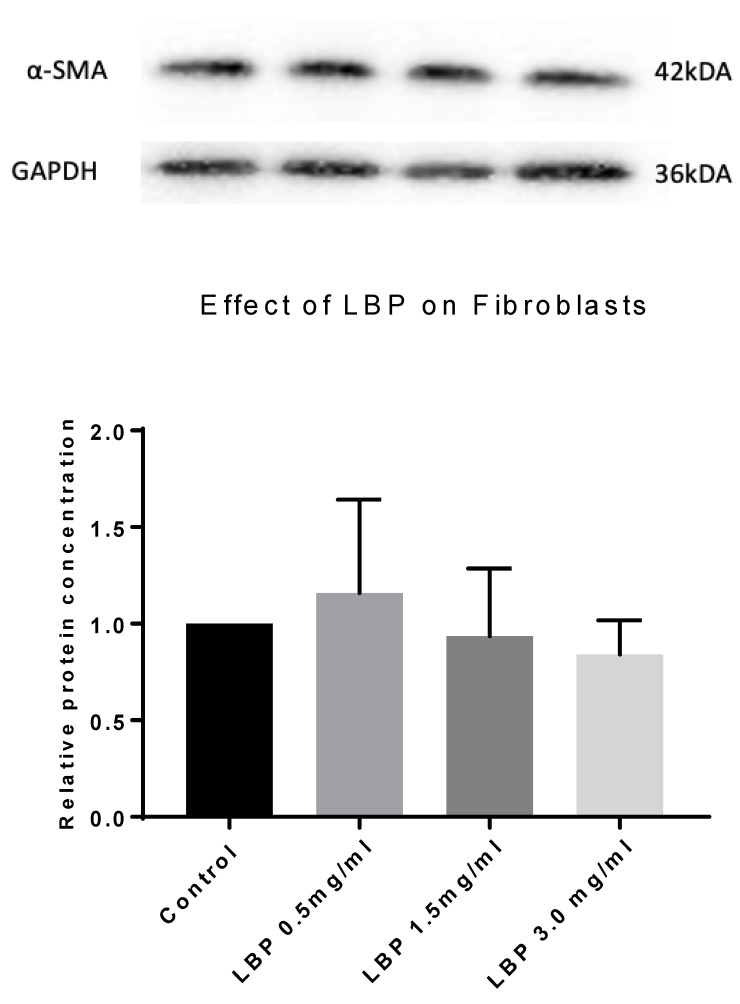
Graph showing α-SMA protein quantification (bottom) with Western blot (top) showing the effect of LBP on αSMA concentration of fibroblasts using one-way ANOVA (*n* = 5 for each group).

Western blot also showed no significant effect of LBP on α-SMA protein concentration confirming the immunofluorescence results (Figure 6). Thus, the effect of LBP is only significant in TGFβ-1 treated cells (Figure 6).

## 4. Discussion

Currently, corneal scarring caused by injuries results in vision loss and significantly impacts the quality of life of patients. Corticosteroid eye drops are used to reduce inflammation and pain after injury but not prevent the occurrence of corneal scarring. Long-term use of corticosteroid is also associated with vision-threatening adverse effects, including cataract and glaucoma. In this study, we identified that pre-treatment of LBP on stromal fibroblasts significantly reduced fibrosis caused by TGFβ1, hence depicting its potential in suppressing myofibroblast differentiation. The reduced α-SMA expression by LBP might suggest a decrease of a-SMA contractile features in wound scarring [18]. At the same time the α-SMA results in significant light scattering observed after injury [19].

The myofibroblasts are the major cells which abnormally produce and deposit extracellular matrix (ECM) proteins such as collagen type I and collagen type III [20,21] which causes disorganized stromal matrix and fibril alignment. The most prominent collagen in the corneal ECM is the tightly packed collagen type I. It is arranged in a highly-organized manner, a feature which essential for maintenance of corneal transparency [22,23]. Myofibroblasts abnormally produce large amounts of ECM deposits alter the composition, organization and mechanical properties of the existing ECM, thus distorting the normal structure and function of the tissue which reduces corneal clarity significantly [24]. Keratocytes have been shown to express collagen type II with TGFβ3 induction [25]. Our model demonstrated that LBP pre-treatment spares collagen type II expression which is important to corneal integrity while reducing the expression of pro-fibrotic collagens specifically collagen type I and collagen type III.

Our experimental model shows that LBP has the ability to affect the viability of myofibroblasts without significantly affecting the fibroblasts which are a fundamental part of corneal wound healing [26]. 

Further work is needed to determine the mechanism of action of LBP. Chien et al. investigated the potential therapeutic effects of LBP extract in dry eye disease. Rats treated with oral LBP extract had a significantly higher Schirmer’s test score and tear break-up time with reduced fluorescein staining compared to other groups in a dose-dependent manner [27,28]. Du et al. assessed the effect of LBP extract in UV-B damaged corneal epithelial cells and found that LBP prevents apoptosis of injured corneal epithelial cells via inhibition of the JNK-Bax-caspase 3 pathway [17]. Xiao et al. assessed the effect of LBP in rats with non-alcoholic steatohepatitis and found that LBP has able to attenuate pro-inflammatory cytokines such as TNFα, IL-1β, cyclooxygenase 2(COX2) and monocyte chemoattractant protein-1 (MCP1) as well as profibrotic factors such as TGFβ-1, α-SMA and Smad 2/4 expression significantly compared to untreated rats with NASH [15]. Another study demonstrated that LBP alleviated carbon tetrachloride (CCl4) induced hepatic fibrosis in rats potentially through the toll-like receptor/nuclear factor-kappa B (NF-kB) pathway [16]. However, the underlying mechanism of which LBP exerts its anti-fibrotic properties remains inconclusive.

Our in vitro model demonstrates the anti-fibrotic potential of LBP extract. One of the major concerns with the use of LBP has been a lack of availability of standardized research-grade LBP formulation [27]. Previous studies have resourced LBP from large-scale nutraceutical companies which offer a large range of LBP purity and sourcing [27]. Moreover, since LBP is a group of polysaccharides, it is possible that its therapeutic effects are attributed to a select few or even a singular active compound. Further studies are planned to understand the mechanism of action of LBP in terms of its anti-fibrotic effects.

## 5. Conclusions

LBP solution demonstrates anti-scarring properties by reducing fibrotic protein synthesis such as α-SMA with a trend of reducing collagen type I and collagen type III expression, though the underlying mechanism is still uncertain. In addition, LBP is does not cause significant toxicity to corneal making it a promising agent for topical use in the eye. However, more work is needed to determine the proper dosing of LBP to exhibit the therapeutic effects without inducing possible cellular toxicity.

## Figures and Tables

**Table 1:** List of antibodies used for immunofluorescence staining.

Primary Antibodies	Company	Catalogue Number	Dilution	Host Species
Anti-vimentin	Abcam	Ab92547	1:500	Rabbit
Anti-fibronectin	Abcam	Ab2413	1:1000	Rabbit
Anti-α-smooth muscle actin	Abcam	ab124964	1:250	Rabbit
Anti-collagen type I	Abcam	ab34710	1:500	Rabbit
Anti-collagen type II	Abcam	ab34712	1:150	Rabbit
Anti-collagen Type III	Abcam	ab6310	1:200	Mouse
Secondary antibodies				
Anti-Rabbit IgG H&L (Alexa Fluor^®^ 594)	Abcam	ab150080	1:500	Goat
Anti-Rabbit IgG H&L (Alexa Fluor^®^ 488)	Abcam	ab150073	1:500	Donkey
Anti-Mouse IgG H&L (Alexa Fluor^®^ 594)	Abcam	ab150108	1:500	Donkey

**Table 2:** List of antibodies used in Western blot.

Primary Antibodies	Company	Model Number	Dilution	Host Species
Anti-α-smooth muscle actin	Abcam	ab124964	1:10,000	Rabbit
Anti-GAPDH	Santa Cruz	sc-32233	1:200	Mouse
Secondary antibodies				
Anti-Rabbit IgG H&L (HRP)	Abcam	ab205718	1:2000	Goat
Anti-Mouse IgG H&L (HRP)	Abcam	ab6728	1:5000	Rabbit

## Data Availability

The authors agree to make all materials, data and associated protocols promptly available to readers without undue qualifications in material transfer agreements.
